# Thermal Degradation and Pyrolysis Kinetics of Alkaline-Pretreated Pinewood under Non-Isothermal Conditions

**DOI:** 10.1021/acsomega.6c00224

**Published:** 2026-09-26

**Authors:** Masoumeh Mohandessi, Qiangu Yan, Zhiyong Cai, Caixia Wan

**Affiliations:** † Department of Chemical and Biomedical Engineering, 14716 University of Missouri, 416 S 6th Street, Columbia, Missouri 65211, United States; ‡ Forest Products Laboratory, USDA Forest Service, One Gifford Pinchot Drive, Madison, Wisconsin 53726, United States

## Abstract

Pinewood (PW) residues are abundant lignocellulosic wastes, but their elevated volatile and oxygenated fractions can complicate pyrolysis by promoting complex devolatilization behavior. This challenge motivates the use of NaOH pretreatment of PW to enhance thermal conversion efficiency. This study investigates the catalytic effect of sodium hydroxide (NaOH) on the pyrolysis kinetics of PW under non-isothermal conditions. Thermogravimetric analysis (TGA) was conducted on untreated PW and samples pretreated with varying concentrations of NaOH (0.2, 0.5, 1, and 2 wt %) at four different heating rates (2.5, 5, 10, and 20 K/min). The results demonstrated that NaOH addition facilitates biomass decomposition by reducing peak pyrolysis temperatures. This catalytic effect was intensified with increasing NaOH concentration. To interpret the observed trends, kinetic modeling was performed using three iso-conversional methods: Friedman, Flynn-Ozawa-Wall (FWO), and Kissinger–Akahira–Sunose (KAS). Thermodynamic parameters of activation were also calculated to complement the kinetic analysis and aid in identifying the most effective NaOH treatment. The modeling results revealed notable reductions in activation energy (*E*_α_) for samples pretreated with 0.2 and 0.5 wt % NaOH, confirming the catalytic role of NaOH in enhancing thermal degradation at lower temperatures potentially by promoting glycosidic bond cleavage.

## Graphical abstract

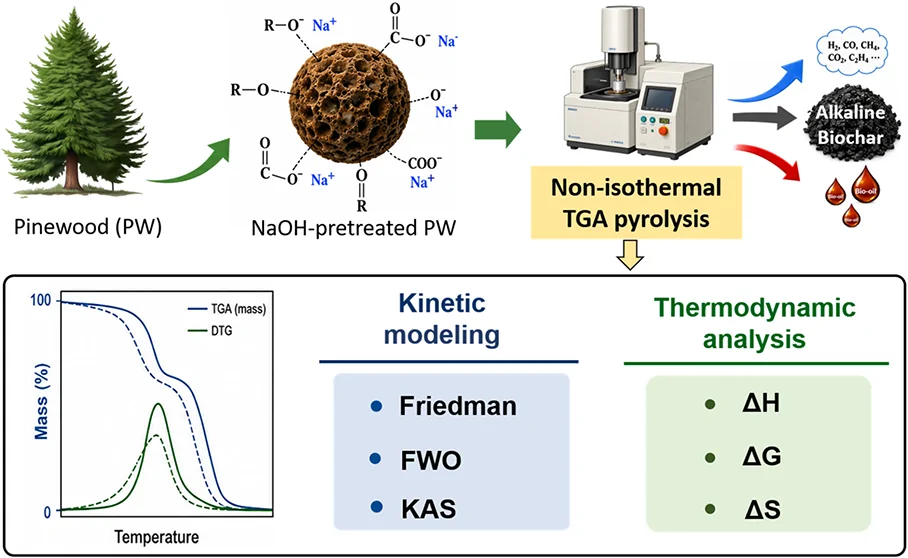

## Introduction

Thermochemical conversion, which transforms biomass directly into energy and fuels, has become a central route for valorizing renewable feedstocks. This pathway includes several processes such as pyrolysis,^1^ gasification,^2^ liquefaction,^3^ and dry distillation.^4^ Among these, pyrolysis has gained particular attention due to its low operating costs, simple operation, and higher value of its byproducts.^5,6^ Pyrolysis refers to thermal decomposition of organic polymers in the absence of oxygen, when heated indirectly at elevated temperatures. Biomass pyrolysis proceeds through evaporation of free moisture followed by primary and secondary thermal degradation, resulting in three primary products, solid biochar, liquid bio-oil, and syngas, with their relative proportions largely influenced by biomass composition.^7,8^ Lignocellulosic biomass is composed of three main structural biopolymers: cellulose, hemicellulose, and lignin.^6,9^ Among these, cellulose decomposes more readily during pyrolysis, while lignin degrades more slowly.^10^ Pinewood (PW) residues are abundant lignocellulosic wastes; however, their relatively high volatile/oxygenated fractions and lignin-rich structure can produce complex devolatilization behavior and shift decomposition toward higher temperatures during pyrolysis. These characteristics motivate converting PW into biochar as a preconditioning step, generating a more carbon-rich and thermally stable intermediate that can be handled and processed more consistently. At the same time, catalytic strategies have been explored to steer thermal pathways and enhance conversion efficiency. Worasuwannarak et al.^11^ investigated the pyrolysis behavior of lignin, cellulose, xylan, and their mixtures, revealing that cross-linking reactions between lignin and cellulose can suppress bio-oil production while promoting char formation. Boot-Handford et al.^12^ investigated wood pyrolysis following treatment with metal chloride salts and found that alkali metal cations catalyzed tar cracking reactions and enhanced the formation of char and gaseous products. Wang et al.^13^ found that even trace additions of alkali and alkaline earth metals to cellulose decreased hydrocarbon yields, with monovalent alkali metals such as Na^+^ and K^+^ exerting a stronger catalytic effect catalytic effect than divalent alkaline earth metals like Mg^2+^ and Ca^2+^.^13^ These studies highlight the sensitivity of biomass thermal decomposition to inorganic additives.^14^ However, how alkali pretreatment modifies the non-isothermal pyrolysis kinetics and associated activation thermodynamic parameters of biomass remains insufficiently resolved, particularly across practical heating programs and catalyst loadings. Alkali pretreatment can alter ash retention and solid-phase chemistry, which introduces practical trade-offs (e.g., mineral accumulation, product distribution shifts) that must be considered when selecting catalyst dosage and operating conditions. Moreover, from an environmental and practical standpoint, catalyzed thermal decomposition is important because reducing the temperature required for devolatilization can lower heat demand during pyrolysis and improve the feasibility of converting woody residues into biochar, bio-oil, and gas under realistic heating programs.

A quantitative understanding of pyrolysis kinetics and thermal degradation behavior is also essential for process design and scale-up,^14^ including mass/energy balance development, reactor sizing, and computational fluid dynamics (CFD) modeling.^15^ The pyrolysis kinetics of biomass generally involve two main stages: the first stage captures the thermal degradation behavior during the reaction, while the second stage involves the determination of kinetic parameters through computational and mathematical modeling approaches.^16^ Thermogravimetric analysis (TGA), combined with model-fitting or model-free methods, is widely employed to quantitatively investigate the kinetic behavior of biomass pyrolysis.^17^ Among these, model-free (iso-conversional) methods are considered more reliable, as they estimate kinetic parameters without assuming a specific reaction mechanism. Slopiecka et al.^18^ conducted a comparative analysis of poplar wood pyrolysis and concluded that the Flynn–Wall–Ozawa (FWO) and Kissinger–Akahira–Sunose (KAS) methods provided more accurate descriptions of biomass decomposition than the Friedman and Kissinger methods. In a related study, Burra and Gupta^19^ applied a first-order distributed activation energy (*E*_α_) model (DAEM) to TGA data to explore synergistic effects in mixed biomass–plastic waste systems. Soria-Verdugo et al.^20^ investigated the pyrolysis kinetics of microalgae using multiple isoconversional methods and found strong agreement among the FWO, KAS, Vyazovkin, and simplified DAEM approaches. Their findings showed that value of model-free methods provided accurate analysis on the kinetic effects of alkali and alkaline earth metals on biomass pyrolysis.

PW residues are abundant lignocellulosic wastes, but their relatively high volatile and oxygenated fractions and lignin-rich structure often lead to complex devolatilization behavior and elevated decomposition temperatures during pyrolysis. Although alkali species are known to affect biomass thermal conversion, the influence of alkaline pretreatment on the non-isothermal pyrolysis kinetics and associated activation thermodynamic parameters of PW during biochar formation remains insufficiently understood. In this study, PW feedstocks pretreated with different NaOH loadings were pyrolyzed under non-isothermal conditions at multiple heating rates, and their thermal degradation behavior was evaluated by TGA/DTG. Model-free iso-conversional methods (Friedman, FWO, and KAS) were used to determine conversion-dependent kinetic parameters, and the corresponding thermodynamic parameters of activation were derived to support interpretation of the pyrolysis kinetics (i.e., the energetics of the rate-limiting transition state). This work establishes a kinetic and thermodynamic basis for evaluating the effects of NaOH loading on PW pyrolysis and for identifying practical alkali loadings for biochar production under non-isothermal conditions. By linking alkali loading with changes in degradation behavior, apparent activation energy, and thermodynamic parameters of activation, the study offers insight that is directly relevant to catalyst selection, thermal process optimization, and energy-efficient conversion of woody residues. The results provide a useful foundation for the design and scale-up of catalytic pyrolysis systems aimed at producing biochar with controlled conversion behavior and improved process feasibility.

## 2 Material and Methods

### 2.1 Biochar Preparation

In this study, PW particle feedstock (40 mesh) was purchased from American Wood Fibers, Inc. (Schofield, WI, USA). Sodium hydroxide (>97%; Fisher Scientific, Hampton, NH, USA) was used as an alkaline pretreatment agent. Pretreatment was conducted at room temperature by immersing PW samples in NaOH solutions of different concentrations, while a deionized water-treated sample served as the control. Each sample was stirred continuously for 2.5 h, followed by 10 min of vacuum treatment to enhance NaOH penetration into the pine particles. The treated samples were then air-dried for 24 h and oven-dried at 105 °C for an additional 24 h. These pretreated samples were subsequently pyrolyzed in a 2-in ceramic tubular reactor (2-inch O.D., 40-inch length, alumina ceramic tube; Richmond, CA, USA). For each run, 30 g of PW particles were packed in the center of the reactor, with both ends filled with quartz wool. The reactor was purged with high-purity argon (99.99%) at 0.2 standard liters per minute (SLPM) for 30 min before heating. Pyrolysis was performed over a temperature range of 350–600 °C at heating rates of 2.5, 5, 10, and 20 K/min, and the final temperature was maintained for 30 min. The NaOH loading range (0, 0.2, 0.5, 1.0, 2.0 wt % of the dried feedstock) was selected to represent practical catalytic impregnation levels rather than high-severity chemical activation. Biochar samples were collected after the reactor cooled to room temperature. The untreated and NaOH-treated PW samples analyzed by TGA prior to fixed-bed pyrolysis and subsequently pyrolyzed for biochar production, were denoted as 0%NaOH + PW, 0.2%NaOH + PW, 0.5%NaOH + PW, 1.0%NaOH + PW, and 2.0%NaOH + PW, respectively. Detailed preparation procedures and related results were reported in Yan et al.^21^ Because higher alkali loadings can increase inorganic retention (ash) and alter char properties and late-stage devolatilization behavior while offering limited additional benefit, 2.0 wt % was selected as the upper limit to capture potential overloading effects.

### 2.2 TGA Analysis

Non-isothermal pyrolysis experiments were conducted using a Mettler Toledo TGA/DSC 3+ thermogravimetric analyzer (Mettler-Toledo, Columbus, OH, USA). Samples were heated from room temperature to 600 °C at four different heating rates: 2.5, 5, 10, and 20 K/min. A plateau at 105 °C was maintained for 20 min prior to heating to ensure the removal of free moisture. Approximately 10 mg of each sample was placed in alumina crucibles for analysis. Throughout the experiments, an inert atmosphere was maintained using a constant nitrogen flow rate of 100 mL/min. The degree of conversion (α) at any given time (*t*) was determined from the initial (i) and final (f) residual masses (TGA, expressed in wt %) according to eq ([Disp-formula eq1])

1 $$\alpha =\frac{{TG}_{i}-{TG}_{t}}{{TG}_{i}-{TG}_{f}}$$

where the conversion degree was set to 0 at the initial decomposition temperature (106 °C) and 100% at the final temperature (600 °C).

### 2.3 Pyrolysis Kinetics

Kinetic parameters are essential for understanding the pyrolytic behavior and underlying reaction mechanisms during biomass pyrolysis. The thermal decomposition of PW can be generally described by the following reaction scheme

2 PW→Volatiles+char+Tar+Water

The pyrolysis reaction rate of PW as a function of temperature can be described by the Arrhenius equation

3 $$\frac{d\alpha }{dt}=A\exp\left(\frac{{-E}_{a}}{\mathrm{RT}}\right){\left(1-\alpha \right)}^{n}$$

where, *A* is the pre-exponential factor (s^–1^), *E*_a_ is the activation energy (kJ/mol), and *n* is the reaction order. *R* denotes the universal gas constant (8.314 J/mol·K), and α represents the conversion degree of the reaction.

At a constant heating rate, the reaction temperature increases linearly with time and can be expressed as

4 $$T=T_{0}+\beta t$$

where *T* and *T*_0_ are the reaction temperature and initial temperature (K), respectively, β represents the heating rate of the sample (K·min^–1^), and *t* is time (min). By substituting eq ([Disp-formula eq4]) into eq ([Disp-formula eq3]), eq 5 is obtained

5 $$\frac{d\alpha }{\mathrm{dT}}=A\exp\left(\frac{{-E}_{a}}{\mathrm{RT}}\right){\left(1-\alpha \right)}^{n}$$

The symbols in eq 5 use the same units and definitions as eq ([Disp-formula eq4]).

By integrating both sides of eq ([Disp-formula eq5]), the general integral form of the reaction rate is obtained as eq ([Disp-formula eq6])

6 $$g\left(\alpha \right)=\int_{0}^{\alpha }\frac{d\alpha }{f\left(\alpha \right)}=\frac{A}{\beta }\int_{T_{0}}^{T}\exp\left(\frac{-E}{\mathrm{RT}}\right)dt\approx \frac{A}{\beta }\int_{0}^{T}\exp\left(\frac{-E}{\mathrm{RT}}\right)dT$$

*g*(α) is the integral conversion function (dimensionless), where α is the degree of conversion (dimensionless) and *f*(α)­is the reaction model function (dimensionless); the temperature integral is evaluated from the initial temperature *T*_0_(K) to the reaction temperature *T* (K) under a constant heating program with β = d*T*/d*t* (K·min^–1^) in this study: 2.5, 5, 10, and 20 K·min^–1^. The Arrhenius parameters are the pre-exponential factor *A* (min^–1^), activation energy *E* (reported as kJ mol^–1^) and the universal gas constant *R* = 8.314 J/mol·K.

Three iso-conversional methods, Friedman, Flynn–Wall–Ozawa (FWO), and Kissinger–Akahira–Sunose (KAS), were employed to determine the *E*_α_ and pre-exponential factor (*A*) associated with the pyrolysis of PW samples subjected to different alkali pretreatment conditions.

Assuming a first-order reaction, the Arrhenius-based expression for the Friedman differential isoconversional method can be written as

7 $$\ln\left(\frac{d\alpha }{dt}\right)=\ln\left(A\left(1-\alpha \right)\right)-\frac{E_{a}}{\mathrm{RT}}$$

α is the conversion degree (dimensionless); therefore, $\frac{d\alpha }{dt}$ is the conversion rate with units of min ^–1^. The first-order model term (1 – α) is dimensionless, and the pre-exponential factor *A* has units’ min^–1^. The activation energy *E*_a_ is reported in kJ mol^–1^, the universal gas constant is *R* = 8.314 J/mol·K, and *T* is the absolute temperature (K); *E*_a_ is used in kJ/mol.

FWO method is a widely used model-free and non-isothermal approach which provides an effective means of evaluating the kinetic parameters of solid-state thermal degradation reactions without assuming a specific reaction mechanism. The kinetic model equation for the FWO method, based on the Arrhenius equation and Doyle’s approximation,^22^ is given by

8 $$\log\beta =\log\frac{{\mathrm{AE}}_{a}}{\mathrm{Rg}\left(\alpha \right)}-2.315-0.4567\frac{E_{a}}{\mathrm{RT}}$$

β is the heating rate (K·min^–1^), *A* is the pre-exponential factor (min^–1^); *E*_a_ is the activation energy (kJ·mol^–1^), *R* is the gas constant (8.314 J/K·mol), *T* is temperature (K), and *g*(α) is dimensionless.

KAS method builds upon the isoconversional approach by applying the Coats–Redfern approximation to enhance the accuracy of kinetic parameter estimation compared to the FWO method. The Arrhenius equation for the KAS method is given by

9 $$\ln\left(\frac{\beta }{T^{2}}\right)=\ln\left(\frac{\mathrm{AR}}{E_{a}g\left(\alpha \right)}\right)-\frac{E_{a}}{\mathrm{RT}}$$

The symbols in the KAS relation (eq 9) use the same units and definitions as eq ([Disp-formula eq8]).

### 2.4 Eyring Activation Parameters

The Friedman, FWO and KAS models were further employed to estimate the thermodynamic parameters of activation for the pyrolysis process based on Eyring transition-state theory. These parameters describe the enthalpy (Δ*H*), Gibbs free energy (Δ*G*), and entropy (Δ*S*) associated with formation of the activated complex (transition state) from the reacting biomass, and therefore characterize the energy barrier that controls the rate of pyrolysis. The enthalpy change (Δ*H*), Gibbs free energy change (Δ*G*), and entropy change (Δ*S*) of activation were calculated as given by eqs ([Disp-formula eq10]), ([Disp-formula eq11]), and ([Disp-formula eq12]), respectively.^23^

10 $$\Delta H=E_{a}-{\mathrm{RT}}_{a}$$

11 $$\Delta G=E_{a}+RT_{m}\ln\left(\frac{T_{m}k_{B}}{\hbar A}\right)$$

12 $$\Delta S=\left(\frac{\Delta H-\Delta G}{T_{m}}\right)$$

Where *T*_m_ is the peak temperature obtained from the derivative thermogravimetry (DTG) curve, *k*_B_ is the Boltzmann constant (1.3806 × 10^–23^ J/K), and ℏ is the Planck constant (6.6261 × 10^–34^ J·s).

The resulting kinetic and thermodynamic parameters (*E*_a_, *A*, Δ*H*, Δ*G*, and Δ*S*) were reported as mean values with standard deviation based on at least three replicates. ANOVA analysis (F-test) was performed using Origin 2022 (9.9.0.225).

## 3 Results and Discussion

### 3.1 Characteristics of Treated and Untreated Pinewood Samples

Proximate analysis (moisture, volatile matter (VM), ash, and fixed carbon (FC)) and ultimate analysis (C, H, O, N, and S; wt %) were determined for raw PW and NaOH-impregnated PW feedstocks prior to pyrolysis and are summarized in Table 1. This baseline characterization is necessary because VM, ash content, and FC govern devolatilization and mineral-catalyzed reactions during thermal treatment, while CHONS ratios (and H/C and O/C) constrain the extent of deoxygenation and structural evolution that ultimately controls the TG/DTG behavior and derived kinetic parameters. Incremental NaOH addition produced a pronounced, dosage-dependent shift in the proximate composition, whereas the bulk elemental composition changed only slightly. Moisture remained nearly constant (5.8–6.1%), indicating that NaOH loading did not measurably alter hygroscopicity. In contrast, VM decreased from 76.5 wt % (0%NaOH + PW) to 69.4 wt % (2.0%NaOH + PW), while FC increased from 16.8 to 21.5 wt %. The concurrent decrease in VM and increase in FC suggests that NaOH promotes dehydration/decarboxylation and facilitates *in situ* cross-linking during heating, thereby suppressing condensable volatiles and favoring solid residue formation.^24^ Ash increased six-fold (0.5 to 3.0 wt %), indicating that sodium was largely retained in the solid phase as inorganic mineral matter, consistent with the formation of Na-rich carbonates and silicates.^25,26^ Consequently, the FC/VM ratio rose from 0.22 to 0.31, implying a reduced tar-forming tendency and enrichment in mineral-associated catalytic sites.^27^

**Table 1:** Proximate and Ultimate Analyses of Raw Pinewood (PW) and NaOH-Impregnated PW Feedstocks Prior to Pyrolysis

analysis (wt %)	0%NaOH + PW	0.2%NaOH + PW	0.5%NaOH + PW	1.0%NaOH + PW	2.0%NaOH + PW
Proximate Analysis					
Moisture content	6.2	5.9	6.0	5.8	6.1
Volatile matter	76.5	75.2	73.2	72.6	69.4
Ash content	0.5	0.9	1.4	1.9	3
Fixed carbon content	16.8	18.1	19.4	19.7	21.5
Ultimate Analysis					
C	51.3	51.9	52.4	50.9	52.1
H	5.6	5.6	5.7	5.7	5.6
O	41.71	41.19	41.64	41.96	40.97
N	1.37	1.29	1.25	1.42	1.31
S	0.02	0.02	0.01	0.02	0.02
H/C molar ratio	1.30	1.28	1.29	1.33	1.28
O/C molar ratio	0.61	0.59	0.59	0.61	0.59
C/N molar ratio	43.68	46.93	48.90	41.81	46.39

Despite these marked proximate shifts, the ultimate analysis varied only modestly because the introduced Na is not quantified in CHONS measurements and is expressed primarily as increased ash rather than incorporation into the organic carbon framework. Carbon increased slightly from 51.3 to 52.4 wt % (up to 0.5 wt % NaOH) before stabilizing, while oxygen decreased marginally from 41.71 to 40.97 wt %, resulting in only a small reduction in O/C (0.61 → 0.59). Hydrogen remained nearly constant (≈5.6–5.7 wt %), keeping H/C near ∼1.3, which indicates that the applied treatment severity was insufficient to drive strong aromatization despite some selective deoxygenation.^28^ The persistently high H/C ratio also suggests substantial residual surface functionality, including basic oxygenated groups that may be introduced or stabilized by NaOH. Together, the simultaneous rise in FC and ash supports a combined effect of a more condensed carbon matrix and embedded sodium-containing species, which can enhance char reactivity (e.g., lower ignition temperature) and provide active sites for secondary reactions such as tar reforming.^29^ Notably, while deoxygenation appears to plateau beyond ∼1 wt % NaOH, ash continues to increase, indicating diminishing returns and potential loss of carbon quality at higher dosages. Overall, 0.5–1.0 wt % NaOH provides a practical balance between mineral loading and carbon quality, whereas 2.0 wt % yields the most mineral-rich, low-volatile material suited for applications that benefit from alkali-promoted catalysis.

### 3.2 Experimental Characterization of the Pyrolysis Behavior of Untreated and Pretreated PW Samples

The thermal decomposition behavior of untreated PW (0%NaOH + PW) and pretreated samples with varying concentrations of NaOH (0%, 0.2%, 0.5%, 1.0%, and 2%) was investigated using thermogravimetric analysis (TGA) at heating rates of 2.5, 5.0, 10, and 20 K/min. The TGA and DTG profiles for all tested samples are illustrated in Figures 1 and S1. Across all conditions, the thermal degradation proceeded through three different stages: initial moisture removal, active devolatilization, and residual char formation. In the first stage, a minor mass loss occurred due to the evaporation of physically adsorbed water. The second stage accounted for the most substantial weight reduction due to the pyrolytic breakdown of organic matter. The final stage is characterized by the progressive degradation of lignin and the formation of thermally stable char residues. This triphasic pattern was consistently observed for both the untreated and alkali-treated samples.^30^

**Figure 1:**
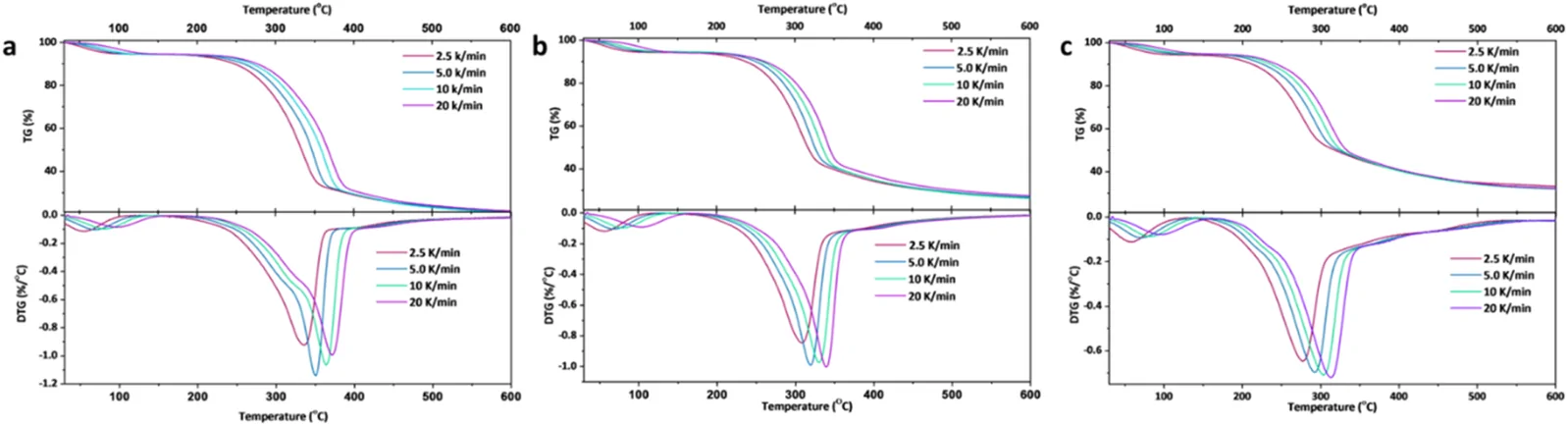
Thermal decomposition behavior of 0%NaOH + PW (a), 0.5%NaOH + PW (b), and 2.0%NaOH + PW (c) at heating rates of 2.5, 5.0, 10.0, and 20.0 K/min showing different peaks of dehydration, devolatilization, and biochar formation.

The thermal degradation of 0%NaOH + PW sample occurred in three distinct stages. The first stage, ranging from approximately 30 to 180 °C, corresponds to the dehydration phase. Within this range, unbound moisture was released below 110 °C, followed by the removal of some extractives and physically bound water between 110 and 180 °C. The second stage, known as the active pyrolysis phase, began from 180 to 420 °C. This zone is marked by the most significant mass loss, primarily due to the thermal decomposition of volatile constituents in the biomass. The third stage started around 420 °C and extended up to approximately 570 °C, where lignin gradually decomposed. In the third thermal degradation zone, lignin decomposition occurred along with various exothermic and endothermic reactions among the remaining organic compounds. This stage, typically referred to as the carbonization and pyrocondensation phase, is characterized by the formation of more stable aromatic structures and cross-linked char residues.^31^ Beyond 570 °C, mass loss plateaued, indicating the near-complete conversion of biomass into solid char, known as the char formation zone. During the active pyrolysis stage, the untreated pine exhibited the highest weight loss. During the active pyrolysis phase, NaOH facilitated the dissociation of interunit linkages, leading to an accelerated release of volatiles and a noticeable reduction in mass. In the final stage, the residual carbonaceous material continued to degrade at a slower rate over a broad temperature range.

Characteristic temperatures for the main devolatilization event were extracted from the TG/DTG curves, including the onset temperature (Tonset), the DTG peak temperature (Tpeak), and the offset temperature (Toffset), together with the associated mass loss in this main stage and the residual mass at the final temperature (Tables S1 and S2). Across all samples, Tonset occurred in the range of ∼167–230 °C and marked the transition from minor low-temperature losses (e.g., remaining bound water and light oxygenates after the 105 °C hold) to the primary devolatilization regime. A dominant DTG maximum was observed at Tpeak (∼278–373 °C depending on sample and heating rate), followed by a high-temperature tail extending to Toffset (∼297–391 °C) that reflects slower decomposition of more thermally stable structures and progressive carbonization.

The heating rate produced a consistent thermal shift in both TG and DTG profiles. Increasing β from 2.5 to 20 K/min shifted Tonset, Tpeak, and Toffset to higher temperatures for every NaOH loading, indicating thermal lag and temperature gradients within particles at faster heating programs. For example, for 0%NaOH + PW, Tpeak increased from 336.4 °C (2.5 K/min) to 372.7 °C (20 K/min), while for 2.0%NaOH + PW, Tpeak increased from 277.7 to 314.0 °C over the same heating-rate range. Despite this temperature shift, the overall DTG shape (a dominant peak plus a trailing shoulder/tail) was preserved, implying that heating rate mainly delays the apparent temperature at which comparable conversion is reached rather than changing the qualitative sequence of degradation events.

NaOH pretreatment produced a clear catalytic effect on the devolatilization temperatures, evidenced by systematic reductions in Tonset, Tpeak, and Toffset relative to the untreated samples at all heating rates. At β = 10 K/min, Tpeak decreased from 363.2 °C for 0%NaOH + PW to 336.9 °C (0.2%NaOH + PW), 330.4 °C (0.5%NaOH + PW), 321.3 °C (1.0%NaOH + PW), and 302.8 °C (2.0%NaOH + PW). A similar downward shift was observed for Tonset (222.6 °C → 194.2 °C from 0% to 2.0% NaOH at 10 K/min) and for Toffset (381.8 °C → 323.8 °C from 0% to 2.0% NaOH at 10 K/min). These reductions indicate that sodium-containing species introduced during impregnation promoted earlier bond scission and accelerated the formation and release of volatile products, thereby lowering the temperatures required to reach the maximum mass-loss rate in the DTG curve.

In parallel with the temperature shifts, NaOH reduced the magnitude of mass loss associated with the main devolatilization stage and increased the remaining solid fraction at the final temperature, consistent with a more mineral-associated and fixed-carbon-rich solid phase after pretreatment. For instance, the main-stage mass loss decreased from ∼60.0–62.3% for 0%NaOH + PW to ∼52.1–55.5% for 0.2%NaOH + PW and ∼49.6–51.5% for 0.5%NaOH + PW across the tested heating rates. This decrease continued at higher NaOH loadings (e.g., ∼47.7–50.5% for 1.0%NaOH + PW and ∼39.5–44.9% for 2.0%NaOH + PW). Correspondingly, the residue at the final temperature increased from ∼20.9–22.2% for 0%NaOH + PW to ∼25.1–26.3% for 0.2%NaOH + PW, ∼26.4–27.7% for 0.5%NaOH + PW, ∼28.5–29.1% for 1.0%NaOH + PW, and ∼31.7–32.9% for 2.0%NaOH + PW. In DTG terms, these trends are consistent with a reduction in the intensity of the main DTG peak (less mass released within the principal devolatilization window) and a comparatively larger retained solid fraction, which aligns with the measured increase in ash/FC with NaOH dosage and supports the interpretation that pretreatment modifies both the reactive organic fraction and the mineral-associated char formation pathway.

### 3.3 Kinetic Analysis

Understanding biomass pyrolysis kinetics through iso-conversional model-free methods is crucial for the effective design and optimization of pyrolysis and gasification reactors for biofuel production. In this study, the Friedman, FWO, and KAS methods were employed to estimate key kinetic and thermodynamic parameters, including *E*_α_, *A*, enthalpy change, entropy change, and Gibbs free energy change. *E*_α_ is a key kinetic parameter in governing the overall reaction mechanism due to the heterogeneous and non-isothermal nature of biomass pyrolysis.^32^

#### 3.3.1 Activation Energy and Pre-Exponential Factors

The *E*_α_ and *A* for the various 0%NaOH + PW and NaOH-pretreated samples were determined from the slope and intercept of the linear regression lines derived from the iso-conversional models, as summarized in Table S3. The variation in average *E*_α_ values obtained from the Friedman, FWO, and KAS methods was found to be ≤1 kJ/mol, which aligns with the guidelines recommended by the ICTAC Kinetics Committee for consistency among model-free methods.^32^ A general increasing trend in *E*_α_ was observed with rising α across all five samples, suggesting a progressive resistance to thermal degradation as pyrolysis proceeds.

As shown in Figure 2, the trend of *E*_α_ as a function of α obtained using the Friedman method consistently exhibited higher values compared to those calculated by the FWO and KAS methods, especially at higher levels of conversion. For instance, at α = 0.90, Friedman gave 321 kJ mol^–1^ for 0%NaOH + PW, whereas the corresponding FWO and KAS averaged 310 and 315 kJ mol^–1^ (a +11 % disparity). This discrepancy was evident across all five samples, with noticeable differences observed both samples obtained from untreated PW (Figure 2a) and pretreated PW with the highest NaOH concentration (Figure 2e). The divergence in *E*_α_ estimates became more pronounced as α increased. This behavior can be attributed to the differential nature of the Friedman method, which is known to be more sensitive to experimental noise and can yield comparatively larger errors, especially at high conversion levels.^20^

**Figure 2:**
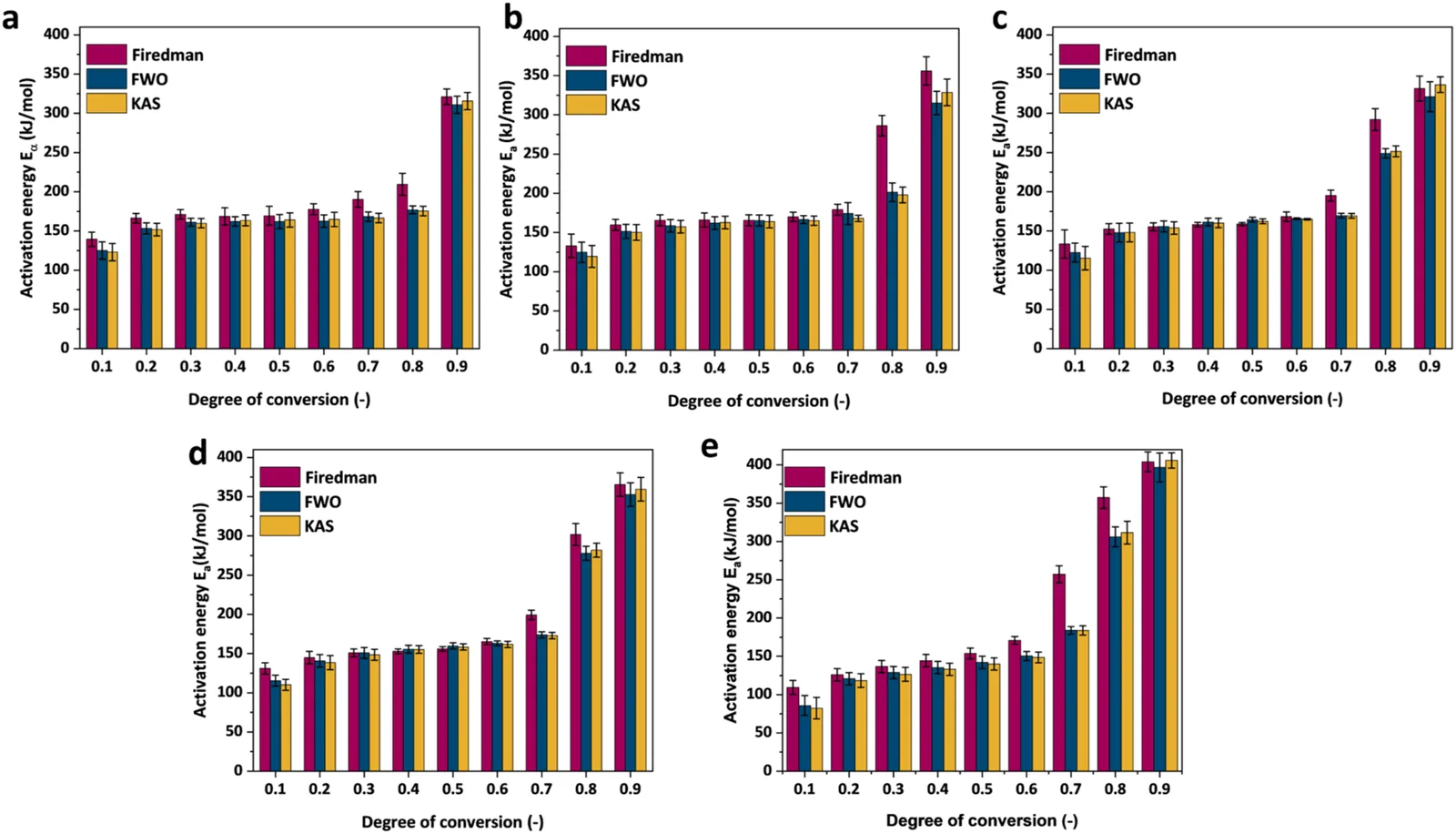
Activation energy value changes with respect to degree of conversion (α) for Friedman, FWO, and KAS models for 0%NaOH + PW (a), 0.2%NaOH + PW (b), 0.5%NaOH + PW (c), 1.0%NaOH + PW (d), and 2.0%NaOH + PW (e).

The *E*_α_ values obtained using the FWO and KAS methods showed good consistency and greater stability compared to those derived from the Friedman method. For both 0%NaOH + PW (Figure 2a) and 0.2%NaOH + PW (Figure 2b), the *E*_α_ obtained from the FWO and KAS models remained nearly constant throughout the principal conversion window (α = 0.20–0.70), varying only from 124 to 168 kJ mol^–1^ for 0%NaOH + PW and 124 to 174 kJ mol^–1^ for 0.2%NaOH + PW in FWO method, and indicating uniform degradation kinetics across the main pyrolysis phase (*p* < 0.05). However, a sharp increase in *E*_α_ was observed as the α approached 0.9, reaching values around 310–315 kJ/mol. This significant rise in *E*_α_ attributed to the decomposition of lignin, which possesses a highly cross-linked and aromatic structure with strong C–C bonds that require higher energy input to break.^7^ For pretreated PW samples impregnated with 0.2 to 0.5 wt % NaOH, the *E*_α_ profiles derived from the FWO and KAS methods remained relatively flat only up to a conversion degree of approximately α of 0.70 (Figure 2b,c). Beyond this point, *E*_α_ increased sharply from around 169 kJ mol^–1^ to over 250 kJ mol^–1^ by α = 0.8. In contrast, for both 0%NaOH + PW and 0.2%NaOH + PW NaOH-treated sample, the sharp increase in *E*_α_ occurred only at later stages of the pyrolysis process, specifically at conversion degrees around α = 0.85–0.90 (Table S1 and Figure 2b). This delayed rise indicates that these samples retained more consistent thermal degradation behavior across a wider conversion range. Moreover, the *E*_α_ profiles reveal that the catalytic influence of NaOH does not increase monotonically with dosage. While low NaOH loadings effectively lower activation barriers within the main devolatilization region, concentrations ≥1 wt % shift the onset of the barrier increase to earlier stages of conversion and elevate the late-stage activation energy. These trends underscore that optimal catalyst loading must be carefully selected rather than assumed to improve simply with increasing NaOH dosage.

The variation in *E*_α_ with increasing conversion degree for all samples, as determined using the Friedman, FWO, and KAS methods, is presented in Figure 3. Across all three is-oconversional models, the *E*_α_ profiles of the 0%NaOH + PW exhibited a consistent trend. An initial rise in *E*_α_ was observed up to α ≈ 0.2, corresponding to the transition from moisture evaporation to the onset of hemicellulose and cellulose decomposition. This was followed by a relatively broad plateau between α ≈ 0.30 and 0.70, during which *E*_α_ remained nearly constant (from 171 to 190 kJ·mol^–1^), indicating a stable degradation phase dominated by the breakdown of the primary polysaccharide components.

**Figure 3:**
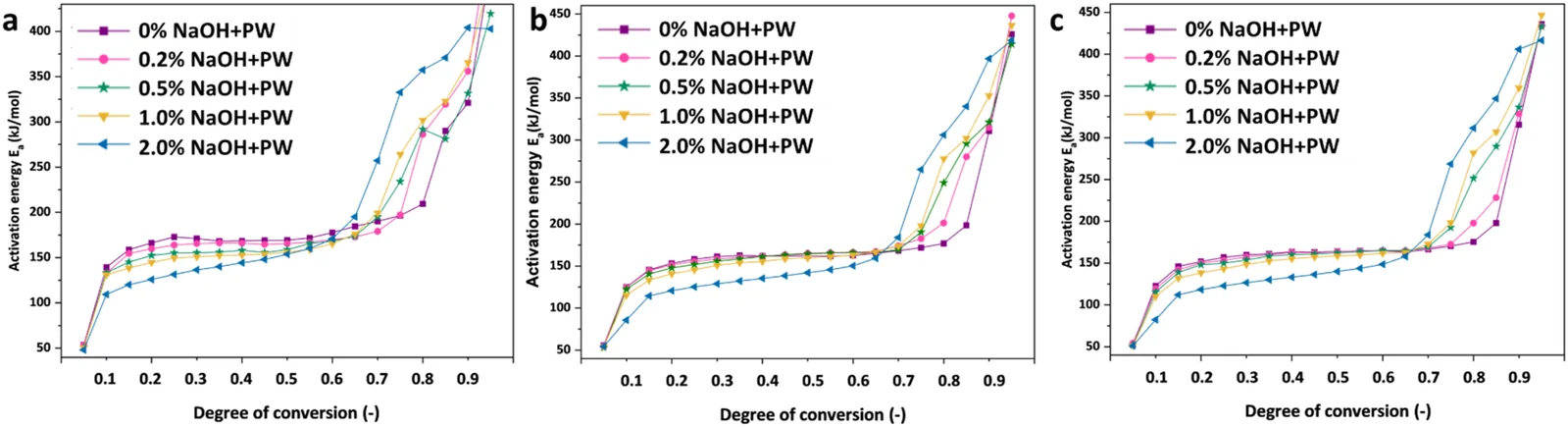
Variation of activation energies of pretreated samples with the extent of conversion for Friedman (a), FWO (b), and KAS (c) models.

In pretreated PW with 2.0 wt % NaOH, the *E*_α_ plateau dropped to as low as 85–150 kJ mol^–1^ over α = 0.1–0.6, compared to 139–177 kJ mol^–1^ for 0%NaOH + PW. However, these catalytic effects were accompanied by an earlier onset of the *E*_α_ surge. This transition occurred earlier than in the untreated sample, where a similar increase was observed only after α = 0.85, indicating a shift of 7–12% in conversion degree. Moreover, the late-stage barrier for 2.0%NaOH + PW exceeded 380, nearly 90 kJ mol^–1^ higher than that of 0%NaOH + PW. This suggests that while lower NaOH concentrations (0.2–0.5 wt %) effectively catalyzed glycosidic bond cleavage and lowered the energy barrier during the main pyrolysis phase, higher concentrations led to an early loss of catalytic advantage. The 0.5 wt % NaOH treatment maintained a minimum plateau of ∼165 kJ mol^–1^ (as determined by FWO and KAS), representing the catalytic optimum without significantly impeding mass transport (*p* < 0.05). This earlier and steeper rise in *E*_α_ at higher NaOH loadings is possibly attributed to the formation of stable Na–O–C complexes, which promote the development of a more condensed, microporous char structure. As a result, the release of volatiles became increasingly diffusion-limited well before full conversion was achieved.^33^

The consistent ordering of *E*_α_ curves across all three kinetic models supports the conclusion that NaOH loadings in the range of 0.2–0.5 wt % define an effective catalytic window.

In addition to *E*_α_, pre-exponential factors were also evaluated and presented alongside in Table S3. This table reports the values of the pre-exponential factor for all samples across various degrees of conversion, as calculated using isoconversional models. The pre-exponential factor plays a crucial role in defining the frequency and likelihood of successful molecular collisions, thereby influencing the overall reaction kinetics. In the context of biomass pyrolysis, *A* values offer important insights into the reaction mechanism and the complexity of the thermochemical conversion process. In model-free kinetic approaches, the pre-exponential factor and *E*_α_ are mathematically related, meaning that variations in *E*_α_ with the extent of pyrolysis are accompanied by corresponding changes in *A*.^34^

For 0%NaOH + PW, the *A* values determined by the Friedman, FWO, and KAS methods ranged from 3 × 10^11±8^ to 1 × 10^20^ ^±6^ s^–1^, 5 × 10^11±15^ to 3 × 10^23^ ^±8^ s^–1^, and 8 × 10^11±14^ to 8 × 10^23±8^ s^–1^, respectively. The broad variation in *A* values across the degree of conversion reflects the inherent structural complexity of the biomass feedstock and the multistep nature of the pyrolysis process.^35^ The pre-exponential factor is influenced by a combination of parameters, including *E*_α_, the universal gas constant, and heating rate. Typically, *A* values greater than 10^9^ s^–1^ are associated with simpler or more reactive systems, whereas values around or below 10^9^ s^–1^ suggest lower reactivity and more complex reaction mechanisms involving condensed-phase intermediates.^36^ In all samples in this study for all conversion rate and three methods, *A* values were more than 10^9^ s^–1^. For example, in 0.5%NaOH + PW, *A* values ranged from 3 × 10^13±4^ to 1 × 10^24±^ ^33^ s^–1^, at α = 0.1 – 0.9, in FWO method.

#### 3.3.2 Thermodynamic Parameters of Activation Analysis

Thermodynamic parameters (Δ*H*, Δ*G*, and Δ*S*) were calculated using eqs ([Disp-formula eq10])–12 and are summarized in Table 2. The enthalpy of activation (Δ*H*) represents the additional energy required for the reacting biomass to reach the activated complex (transition state) during pyrolysis, i.e., the energy barrier associated with the rate-limiting bond redistribution, rather than the total energy consumed to form the final solid, liquid, and gaseous products (char, bio-oil, and syngas).^38^ At low to mid conversion (α = 0.10–0.60), NaOH pretreatment generally reduced Δ*H* compared with 0%NaOH + PW. In the KAS method at α = 0.10, Δ*H* decreased from 118 kJ/mol to 114–105 kJ/mol for 0.2–1.0 wt % NaOH pretreatment and further to 78 kJ/mol for the 2.0 wt % sample (Figures 4c and S2). Similarly, at α = 0.60, ΔH decreased from 159 kJ/mol for 0%NaOH + PW to 143 kJ/mol for the 2.0 wt % NaOH pretreatment sample (*p* < 0.05). These results indicate a lower energy requirement during the main decomposition stage. In contrast, at high conversion (α ≥ 0.80) the NaOH-treated samples exhibited substantially higher ΔH than the untreated sample. For instance, in the KAS method, Δ*H* at α = 0.80 increased from 169 kJ/mol for 0%NaOH + PW to 192–306 kJ/mol for the treated samples, showing that the late-stage decomposition became more energy demanding, particularly at higher NaOH loading.^37^ This suggests that, as pyrolysis progressed and conversion increased, the interactions among biomass constituents became more pronounced due to the increasing complexity of bond dissociation and rearrangement during thermal degradation. A similar trend was observed using the Friedman and FWO methods. Among the three models, the Friedman method consistently yielded higher enthalpy values. For example, for 0%NaOH + PW, Δ*H* increased from 134 to 315 kJ/mol in the Friedman method, from 120 to 305 kJ/mol in the FWO method, and from 118 to 304 kJ/mol in the KAS method over the conversion range of 0.1 to 0.9.

**Figure 4:**
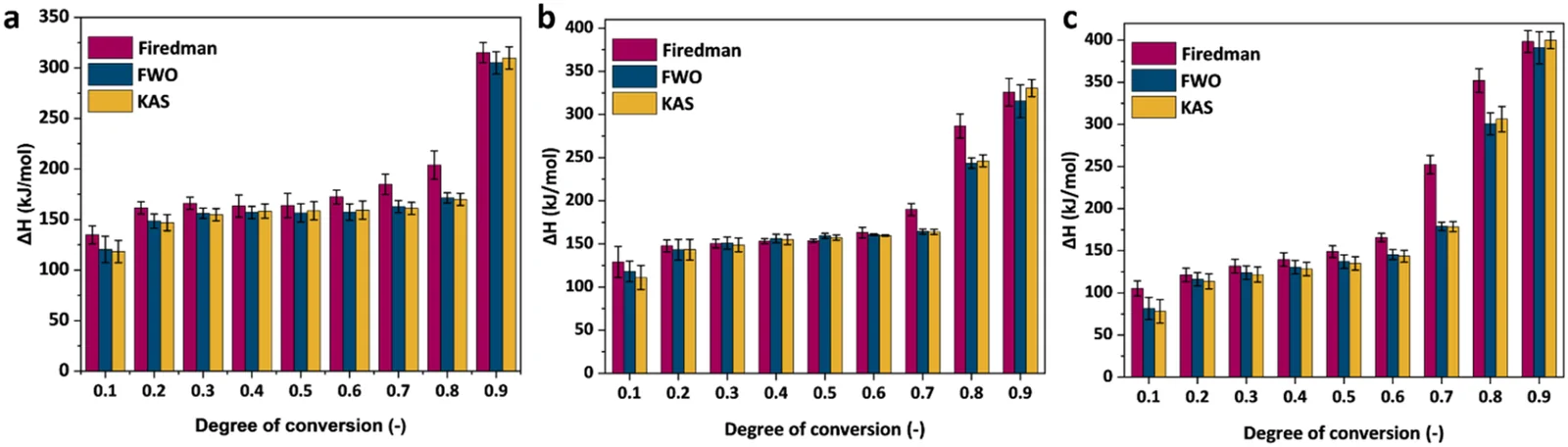
Enthalpy (Δ*H*) value changes with respect to degree of conversion (α) for Friedman, FWO and KAS models for 0%NaOH + PW (a), 0.5%NaOH + PW (b), and 2.0%NaOH + PW (c).

**Table 2:** Thermodynamic Parameters of Activation using the Friedman, FWO, and KAS Models

	0%NaOH + PW			0.2%NaOH + PW			0.5%NaOH + PW			1.0%NaOH + PW			2.0%NaOH + PW		
α	Δ*H* (kJ/mol)	Δ*G* (kJ/mol)	Δ*S* (J/K·mol)	Δ*H* (kJ/mol)	Δ*G* (kJ/mol)	Δ*S* (J/K·mol)	Δ*H* (kJ/mol)	Δ*G* (kJ/mol)	Δ*S* (J/K·mol)	Δ*H* (kJ/mol)	Δ*G* (kJ/mol)	Δ*S* (J/K·mol)	Δ*H* (kJ/mol)	Δ*G* (kJ/mol)	Δ*S* (J/K·mol)
Friedman															
0.1	134 ± 9	154 ± 12	–36 ± 7	128 ± 15	146 ± 15	–33 ± 6	129 ± 18	154 ± 10	–50 ± 7	126 ± 7	151 ± 12	–47 ± 14	105 ± 9	156 ± 15	–103 ± 13
0.2	161 ± 6	186 ± 12	–43 ± 8	155 ± 7	174 ± 8	–33 ± 7	147 ± 7	164 ± 7	–30 ± 4	140 ± 8	163 ± 14	–40 ± 10	121 ± 8	165 ± 11	–81 ± 16
0.3	166 ± 6	191 ± 9	–41 ± 5	160 ± 7	178 ± 7	–30 ± 4	150 ± 5	168 ± 5	–31 ± 3	146 ± 5	166 ± 5	–34 ± 4	131 ± 8	169 ± 19	–67 ± 19
0.4	163 ± 11	198 ± 16	–56 ± 9	160 ± 9	181 ± 10	–34 ± 5	153 ± 3	171 ± 3	–30 ± 1	147 ± 3	168 ± 3	–35 ± 3	139 ± 8	172 ± 10	–56 ± 11
0.5	163 ± 12	202 ± 16	–60 ± 6	160 ± 7	183 ± 8	–37 ± 8	153 ± 2	160 ± 5	–10 ± 2	150 ± 3	170 ± 3	–32 ± 1	148 ± 7	175 ± 14	–44 ± 10
0.6	172 ± 7	199 ± 11	–42 ± 5	164 ± 6	185 ± 6	–33 ± 3	163 ± 6	153 ± 9	17 ± 5	160 ± 4	173 ± 5	–20 ± 5	165 ± 5	177 ± 6	–19 ± 8
0.7	184 ± 10	201 ± 17	–25 ± 3	173 ± 7	188 ± 11	–23 ± 5	189 ± 7	158 ± 12	52 ± 7	194 ± 6	177 ± 16	28 ± 4	252 ± 11	183 ± 17	115 ± 12
0.8	203 ± 14	205 ± 11	–2 ± 1	280 ± 13	196 ± 16	133 ± 11	286 ± 14	185 ± 15	161 ± 12	296 ± 14	191 ± 18	167 ± 13	352 ± 14	241 ± 16	177 ± 10
0.9	315 ± 10	467 ± 10	–22 ± 4	350 ± 18	223 ± 20	183 ± 17	325 ± 16	210 ± 12	166 ± 15	359 ± 15	217 ± 18	207 ± 17	398 ± 13	225 ± 14	248 ± 12
FWO															
0.1	120 ± 13	138 ± 16	–33 ± 3	120 ± 14	144 ± 13	–45 ± 5	118 ± 12	117 ± 12	1 ± 0.1	111 ± 7	112 ± 11	–1 ± 0.1	81 ± 13	129 ± 18	–96 ± 11
0.2	148 ± 7	150 ± 11	–2 ± 0.5	146 ± 9	144 ± 11	4 ± 1	143 ± 12	133 ± 12	17 ± 5	135 ± 8	136 ± 13	0.17	116 ± 8	140 ± 13	–43 ± 4
0.3	156 ± 5	155 ± 6	1 ± 0.5	153 ± 8	149 ± 8	7 ± 1	150 ± 7	144 ± 18	12 ± 2	146 ± 7	145 ± 9	1 ± 0.1	124 ± 8	145 ± 12	–37 ± 16
0.4	156 ± 6	160 ± 11	–4 ± 1	157 ± 8	153 ± 8	6 ± 1	156 ± 5	151 ± 5	8 ± 2	150 ± 5	149 ± 6	1 ± 0.2	130 ± 8	148 ± 8	–31 ± 6
0.5	156 ± 9	165 ± 10	–13 ± 2	160 ± 7	158 ± 8	3 ± 0.5	159 ± 3	153 ± 9	10 ± 3	154 ± 4	154 ± 4	1 ± 0.1	137 ± 8	151 ± 15	–23 ± 2
0.6	157 ± 8	166 ± 9	–13 ± 2	161 ± 5	160 ± 6	1 ± 0.3	160 ± 1	148 ± 1	21 ± 1	158 ± 3	156 ± 3	2 ± 0.5	145 ± 6	153 ± 6	–13 ± 4
0.7	162 ± 6	168 ± 13	–7 ± 3	168 ± 14	163 ± 10	10 ± 1	164 ± 3	139 ± 3	40 ± 3	167 ± 4	157 ± 5	1 ± 0.1	179 ± 5	155 ± 6	40 ± 5
0.8	171 ± 5	170 ± 5	2 ± 4	195 ± 12	164 ± 11	49 ± 7	243 ± 6	161 ± 8	132 ± 9	272 ± 9	159 ± 9	180 ± 5	300 ± 13	158 ± 18	226 ± 10
0.9	305 ± 11	173 ± 16	19 ± 7	309 ± 15	201 ± 15	155 ± 6	315 ± 19	467 ± 12	–219 ± 18	346 ± 15	174 ± 16	250 ± 10	390 ± 19	172 ± 11	314 ± 17
KAS															
0.1	118 ± 11	134 ± 13	–29 ± 4	114 ± 14	137 ± 14	–41 ± 3	111 ± 14	108 ± 14	7 ± 4	105 ± 7	104 ± 8	3 ± 0.8	78 ± 14	124 ± 18	–94 ± 13
0.2	146 ± 8	146 ± 9	1 ± 0.5	145 ± 10	140 ± 10	8 ± 3	143 ± 12	131 ± 11	22 ± 6	133 ± 9	131 ± 10	4 ± 0.6	113 ± 9	135 ± 17	–40 ± 7
0.3	154 ± 6	151 ± 6	5 ± 1	152 ± 8	146 ± 13	11 ± 2	148 ± 8	139 ± 12	16 ± 5	143 ± 7	140 ± 7	5 ± 0.4	121 ± 9	141 ± 10	–34 ± 6
0.4	158 ± 7	159 ± 9	–0.95 ± 0.2	157 ± 8	152 ± 14	9 ± 1	155 ± 6	148 ± 12	11 ± 4	150 ± 5	147 ± 5	5 ± 0.6	128 ± 8	144 ± 17	–28 ± 4
0.5	158 ± 9	166 ± 9	–11 ± 1	158 ± 8	155 ± 8	7 ± 1	157 ± 3	149 ± 6	14 ± 5	153 ± 4	151 ± 4	4 ± 0.7	135 ± 8	147 ± 11	–20 ± 4
0.6	159 ± 9	166 ± 9	–10 ± 2	159 ± 6	157 ± 6	4 ± 1	159 ± 1	144 ± 1	25 ± 1	156 ± 4	153 ± 4	6 ± 0.8	143 ± 7	150 ± 10	–10 ± 3
0.7	161 ± 6	164 ± 12	–5 ± 1	162 ± 4	159 ± 4	5 ± 1	164 ± 3	136 ± 3	45 ± 2	167 ± 4	155 ± 4	2 ± 1	178 ± 6	152 ± 6	45 ± 3
0.8	169 ± 6	166 ± 6	5 ± 1	192 ± 10	162 ± 13	48 ± 5	246 ± 7	159 ± 7	138 ± 4	276 ± 9	159 ± 18	187 ± 14	306 ± 15	159 ± 15	234 ± 11
0.9	309 ± 11	174 ± 12	198 ± 7	327 ± 17	165 ± 18	228 ± 17	330 ± 10	238 ± 18	133 ± 7	353 ± 15	176 ± 8	257 ± 12	400 ± 10	175 ± 5	322 ± 11

Across all samples and kinetic models (Friedman, FWO, and KAS), the average difference between *E*_α_ and Δ*H* was small (approximately 4–5 kJ/mol; Tables 2 and S3), consistent with the formation of an activated complex during thermal degradation.^39^ For example, for 0.5%NaOH + PW at α = 0.7 in the KAS method, *E*_α_ and Δ*H* were 169 and 164 kJ/mol, respectively. Similar behavior was reported by Kaur et al.^40^ The close agreement between *E*_a_ and Δ*H* values suggests efficient thermochemical conversion and points to the formation of relatively simple pyrolysis products.^41^

Δ*G* represents the free-energy barrier associated with the formation of the activated complex during pyrolysis and reflects the energy required for the reaction to proceed. The Δ*G* values at different degrees of conversion are presented in Table 2 and Figure S3. At low to mid conversion, NaOH pretreatment generally reduced Δ*G* relative to the untreated PW sample, indicating a more energetically favorable progression during the main decomposition stage. For example, at α = 0.70, Δ*G* in the Friedman method decreased from 201 kJ/mol for the untreated sample to 188, 158, 177, and 183 kJ/mol for the 0.2, 0.5, 1.0, and 2.0 wt % NaOH pretreatment levels, respectively. A similar pattern was observed in the FWO and KAS methods, where Δ*G* at α = 0.70 decreased from 168 to 155 kJ/mol and from 164 to 152 kJ/mol, respectively (*p* < 0.05). However, at α = 0.90, the behavior became more stage-dependent. In the Friedman method, Δ*G* for the untreated sample increased sharply to 467 kJ/mol, whereas the pretreated samples ranged from 210 to 225 kJ/mol. An increase in Δ*G* with increasing α during pyrolysis indicates greater energy retention within the system, often associated with complex structural rearrangements.^23^ Similar trends have been reported in previous studies involving agricultural waste biomass, which also found consistent or slightly varying Δ*G* values.^42^ Among the three kinetic models, the Friedman method generally yielded the highest Δ*G* values, while the FWO and KAS methods gave relatively close results.^43^ The positive Δ*G* values of activation observed across all stages reflect a substantial free-energy barrier to reaching the activated complex, consistent with the need for continuous external heat input to sustain the reaction rate;^44^ this reflects the kinetics of pyrolysis rather than the overall thermodynamic spontaneity of PW conversion to its final products.

The Δ*S* reflects the formation of new species and the degree of molecular disorder due to three thermal degradations of biomass.^45^ Negative Δ*S* values indicate the formation of a more ordered activated complex, whereas positive values suggest a more disordered transition state with greater molecular freedom. At low to mid conversion (α = 0.10–0.60), the untreated PW sample generally showed negative or near-zero Δ*S* values, while NaOH pretreatment tended to increase Δ*S* and, in some cases, shifted it from negative to positive values (Figure 5). For example, in the Friedman method at α = 0.60, Δ*S* changed from −42 J/K·mol for the untreated PW sample to −33, 17, −20, and −19 J/K·mol for the 0.2, 0.5, 1.0, and 2.0 wt % NaOH pretreatment levels, respectively (*p* < 0.05). Similar tendencies were observed in the FWO and KAS methods, where the 0.5 and 1.0 wt % pretreatment levels often produced positive Δ*S* values earlier in the conversion range. At high conversion, the influence of NaOH pretreatment became much more pronounced. In the Friedman method at α = 0.80, Δ*S* increased from −2 J/K·mol for the untreated sample to 133, 161, 167, and 177 J/K·mol for the 0.2, 0.5, 1.0, and 2.0 wt % pretreated samples, respectively. A comparable increase was observed in the KAS method, where Δ*S* rose from 5 J/K·mol for the untreated sample to 48, 138, 187, and 234 J/K·mol for the pretreated samples at the same conversion level. These results indicate that the later stages of pyrolysis involved the formation of a progressively more disordered activated complex, likely due to intensified bond cleavage and structural rearrangement as conversion approached completion. Across all three kinetic models, the same overall pattern was observed, with NaOH pretreatment generally increasing Δ*S*, especially in the high-conversion region, although the magnitude of the increase varied among methods. Overall, the Friedman method showed the widest variation in Δ*S*, whereas the FWO and KAS methods were generally more similar over most of the conversion range. The coexistence of both positive and negative entropy values across different samples and conversion stages highlights the complex nature of biomass pyrolysis, which involves multiple parallel and sequential reactions yielding a variety of products.^46^ Overall, based on the data presented in Tables 2 and S3, the 0.2% and 0.5%NaOH + PW samples exhibited lower Δ*S*, accordingly ranged from −41 to 5 J/K·mol and 7 to 45 J/K·mol, at α = 0.10–0.70 in KAS method which make them more energy-efficient and economically viable candidates for biochar production under catalytic pyrolysis conditions.

**Figure 5:**
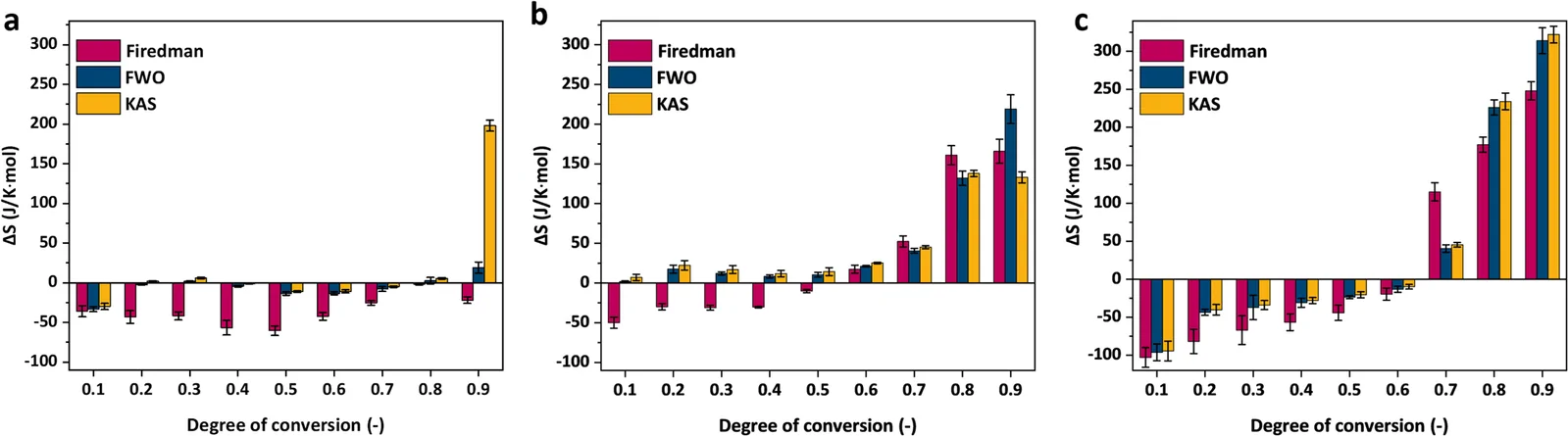
Entropy (Δ*S*) value changes with respect to degree of conversion (α) for Friedman, FWO and KAS models for 0%NaOH + PW (a), 0.5%NaOH + PW (b), and 2.0%NaOH + PW (c).

Table 3 compares the *E*_α_ and thermodynamic parameters obtained in the present study with those reported for other agricultural residues. The results indicate that the selected PW biomass exhibited *E*_α_ values comparable to or better than other crop-based feedstocks. Notably, PW treated with low concentrations of NaOH demonstrates reduced *E*_α_, which is confirmed its potential as an energy-efficient and economically viable option for biochar production. Moreover, understanding thermodynamic parameters such as enthalpy, entropy, and Gibbs free energy is crucial for the effective design of pyrolysis and gasification systems in energy balance assessments. The data generated through TGA/DTG analysis in this study provide essential insights for guiding the development and optimization of thermochemical conversion processes.

**Table 3:** Activation Energy and Thermodynamic Parameters of NaOH Treated Pinewood and Other Agricultural Waste

				thermodynamic properties			
agricultural waste	treated	method	activation energy *E*_α_	Δ*H* (kJ/mol)	Δ*G* (kJ/mol)	Δ*S* (J/K·mol)	refs.
Pine wood	NaOH (0.2%)	FWO	124.7–174.1	120.3–168.8	144–163	–45.1–10.1	this work
		KAS	119.3–169.7	114.9–162.6	137–159	–41.5–5.75	
Wheat straw	-	FWO	165.2–195.2	124.3–155.6	125.1–154.2	–60.25–3.33	^15^
		KAS	167.3–193.6	121.2–150.6	120.2–140.4	–65.25–4.48	
Beech wood	NaCl	FWO	125.4–137.2	-	-	-	^47^
		KAS	122.9–134.1				
Beech wood	KCl	FWO	128.6–137.3	-	-	-	^47^
		KAS	126.3–134.3				
Beech wood	MgCl_2_	FWO	134.4–152.7	-	-	-	^47^
		KAS	133.0–150.1				
Bamboo	-	FWO	173.8–71.1	165.4–62.7	299.8–307.3	–132.9–241.9	^48^
		KAS	174.5–66.5	166.1–58.11	299.8–307.9	–132.14–247.0	

In general, the observed reductions in peak pyrolysis temperature and the lower *E*_α_ values at low NaOH loadings (0.2–0.5 wt %) suggest an operational window where thermal conversion may be achieved with reduced energy input, supporting energy-efficient processing of PW-derived biochar under non-isothermal heating. Future work should (i) quantify product yields and compositions (bio-oil/gas/char) under the fixed-bed conditions used here to link kinetic shifts to practical outcomes, (ii) evaluate catalyst fate and solid-phase mineral accumulation (e.g., ash increase at higher NaOH loadings) and its implications for downstream handling and reactor operation, and (iii) validate the identified low-loading window across additional woody and agricultural feedstocks and broader heating programs to support transferability and scale-up.

## 4 Conclusion

The pyrolysis behavior of PW and samples impregnated with varying concentrations of NaOH were investigated at four different heating rates using thermogravimetric analysis. Thermogravimetric analysis at four heating rates showed that NaOH impregnation catalyzed PW pyrolysis, shifting decomposition to lower temperatures and reducing peak pyrolysis temperatures, with stronger effects at higher NaOH loadings. A model-free iso-conversional kinetic analysis was performed using Friedman, FWO, and KAS methods to estimate *E*_α_ and the pre-exponential factor. FWO and KAS produced consistent activation energy trend and that low NaOH loadings (0.2–0.5 wt %) notably reduced *E*_α_, confirming improved pyrolysis reactivity under mild pretreatment. Furthermore, by coupling iso-conversional models with model-fitting approaches, it was possible to simulate the conversion behavior of each sample as a function of temperature, regardless of the applied heating rate. Higher pre-exponential factors derived from the iso-conversional models suggested increased reactivity of the system at elevated temperatures. Analysis of the thermodynamic parameters of activation yielded positive Δ*H* and Δ*G* over the conversion range, reflecting a substantial energy barrier to the activated complex that requires continuous external heat input to sustain the reaction rate, while the small *E*_α_–Δ*H* difference is consistent with a relatively simple, single-step-like activation process. These findings characterize the kinetics of the rate-limiting step of pyrolysis.

## Supplementary Material



## Notes

Pyrolysis characteristic parameters of the main stages for 0%NaOH + PW, 0.2%NaOH + PW, and 0.5%NaOH + PW samples (Table S1); pyrolysis characteristic parameters of the main stages for 1.0%NaOH + PW and 2.0%NaOH + PW samples (Table S2); kinetic properties obtained from the Friedman, FWO, and KAS models (Table S3); thermal decomposition behavior of 0.2%NaOH + PW and 1.0%NaOH + PW at heating rates of 2.5, 5.0, 10.0, and 20.0 K/min (Figure S1); enthalpy (Δ*H*) changes with respect to degree of conversion (α) for the Friedman, FWO, and KAS models (Figure S2); Gibbs free energy (Δ*G*) changes with respect to degree of conversion (α) for all pretreated samples (Figure S3); entropy (Δ*S*) changes with respect to degree of conversion (α) for the Friedman, FWO, and KAS models (Figure S4) (PDF)
