# Supplementary material for: Thermal Degradation and Pyrolysis Kinetics of Alkaline-Pretreated Pinewood under Non-Isothermal Conditions
Source: ACS Omega. 2026 Sep 26;11(39):58572–83. doi: 10.1021/acsomega.6c00224 (PMC13647414; doi:10.1021/acsomega.6c00224)
Supplement: Supplementary file 1 [file ao6c00224_si_001.pdf]

Supporting Information

Thermal Degradation and Pyrolysis Kinetics of Alkaline-Pretreated Pinewood under Non-Isothermal Conditions

Masoumeh Mohandessi [a, §], Qiangu Yan [b, §, *], Zhiyong Cai [b], Caixia Wan [a,*]

[a] Department of Chemical and Biomedical Engineering, University of Missouri, Columbia, MO 65211, USA

[b] Forest Products Laboratory, USDA Forest Service, Madison, WI 53726, USA

[§] These authors contributed equally.

[*] Corresponding authors. Qiangu Yan: Tel +1 608 231 9501; Email: Qiangu.Yan@usda.gov. Caixia Wan: Tel +1 573 884 7882; E-mail: wanca@missouri.edu.

Number of pages: 8
Number of tables: 3
Number of figures: 4

Table S1. Pyrolysis characteristic parameters of the main stages for 0%NaOH+PW, 0.2%NaOH+PW, and 0.5%NaOH+PW samples.

	**0%NaOH+PW**				**0.2%NaOH+PW**				**0.5%NaOH+PW**			
**β (K/min)**	**2.5**	**5**	**10**	**20**	**2.5**	**5**	**10**	**20**	**2.5**	**5**	**10**	**20**
T onset (°C)	206.46	218.56	222.60	228.92	198.40	212.22	218.27	230.36	204.16	209.34	217.12	219.71
T peak (°C)	336.44	350.26	363.21	372.71	316.15	326.51	336.87	348.96	307.08	319.17	330.40	339.04
T offset (°C)	355.87	364.51	381.78	391.28	333.13	343.50	357.31	369.40	328.53	335.43	347.53	354.43
Mass loss (%)	60.04	60.35	62.25	61.63	52.08	52.39	53.96	55.53	49.57	50.82	51.45	50.81
Residue at final T (%)	21.85	21.53	20.9	22.17	25.39	25.39	26.33	25.07	27.06	27.37	26.43	27.69

Table S2. Pyrolysis characteristic parameters of the main stages for 1.0%NaOH+PW and 2.0%NaOH+PW samples.

	**1.0%NaOH+PW**				**2.0%NaOH+PW**			
**β (K/min)**	**2.5**	**5**	**10**	**20**	**2.5**	**5**	**10**	**20**
T onset (°C)	186.60	193.51	205.60	211.65	167.46	180.41	194.23	205.46
T peak (°C)	296.28	309.24	321.33	328.24	277.71	292.40	302.76	313.99
T offset (°C)	317.01	328.24	342.06	349.83	297.00	312.55	323.78	334.14
Mass loss (%)	47.69	47.68	49.88	50.51	39.53	42.04	43.29	44.85
Residue at final T (%)	29.10	29.10	28.79	28.47	32.92	32.60	31.66	31.98

Table S3. Kinetic parameters determined using the Friedman, FWO, and KAS models.

α	0%NaOH+PW		0.2%NaOH+PW		0.5%NaOH+PW		1.0%NaOH+ PW		2.0%NaOH+PW	
	E_a (kJ/mol)	A (S^{-1})	E_a (kJ/mol)	A (S^{-1})	E_a (kJ/mol)	A (S^{-1})	E_a (kJ/mol)	A (S^{-1})	E_a (kJ/mol)	A (S^{-1})
**Friedman**										
0.1	139±9	$3\times10^{11\pm8}$	132±15	$5\times10^{11\pm37}$	133±18	$7\times10^{10\pm18}$	131±7	$1\times10^{11\pm5}$	109±9	$1\times10^{8\pm10}$
0.2	166±6	$1\times10^{11\pm4}$	159±7	$5\times10^{11\pm5}$	152±7	$8\times10^{11\pm5}$	144±8	$3\times10^{11\pm6}$	125±8	$1\times10^{9\pm7}$
0.3	171±6	$2\times10^{11\pm3}$	165±7	$8\times10^{11\pm5}$	155±5	$7\times10^{11\pm3}$	150±5	$5\times10^{11\pm3}$	136±8	$1\times10^{10\pm6}$
0.4	168±11	$3\times10^{10\pm9}$	165±9	$5\times10^{11\pm7}$	158±3	$8\times10^{11\pm2}$	152±3	$4\times10^{11\pm2}$	144±8	$3\times10^{10\pm5}$
0.5	169±12	$2\times10^{10\pm11}$	165±7	$3\times10^{11\pm4}$	158±2	$1\times10^{13\pm1}$	155±3	$6\times10^{11\pm1}$	153±7	$1\times10^{11\pm4}$
0.6	177±7	$2\times10^{11\pm4}$	169±6	$6\times10^{11\pm3}$	168±6	$2\times10^{14\pm4}$	165±4	$2\times10^{12\pm2}$	170±5	$3\times10^{12\pm3}$
0.7	190±10	$1\times10^{12\pm8}$	179±7	$2\times10^{12\pm4}$	195±7	$1\times10^{16\pm4}$	199±6	$1\times10^{15\pm3}$	257±11	$3\times10^{19\pm9}$
0.8	209±14	$2\times10^{13\pm15}$	286±13	$3\times10^{20\pm8}$	291±14	$9\times10^{21\pm14}$	301±14	$1\times10^{22\pm5}$	357±14	$6\times10^{22\pm15}$
0.9	321±10	$1\times10^{20\pm6}$	356±18	$1\times10^{23\pm14}$	331±16	$1\times10^{22\pm15}$	365±15	$2\times10^{24\pm16}$	404±13	$3\times10^{26\pm28}$
**FWO**										
0.1	124±11	$5\times10^{11\pm15}$	124±13	$1\times10^{11\pm32}$	122±12	$3\times10^{13\pm4}$	115±7	$2\times10^{13\pm5}$	85±13	$2\times10^{8\pm36}$
0.2	153±7	$2\times10^{13\pm5}$	151±9	$5\times10^{13\pm8}$	147±12	$2\times10^{14\pm17}$	140±8	$3\times10^{13\pm7}$	120±8	$1\times10^{11\pm8}$
0.3	161±5	$4\times10^{13\pm3}$	158±8	$8\times10^{13\pm6}$	155±7	$1\times10^{14\pm5}$	150±7	$4\times10^{13\pm5}$	128±8	$3\times10^{11\pm7}$
0.4	162±6	$2\times10^{13\pm4}$	162±8	$7\times10^{13\pm5}$	161±5	$9\times10^{13\pm3}$	155±5	$4\times10^{13\pm3}$	135±8	$7\times10^{11\pm6}$
0.5	161±9	$6\times10^{12\pm6}$	165±7	$5\times10^{13\pm5}$	164±3	$1\times10^{14\pm2}$	159±4	$3\times10^{13\pm2}$	142±8	$1\times10^{12\pm5}$
0.6	162±8	$7\times10^{12\pm6}$	166±5	$4\times10^{13\pm3}$	165±1	$4\times10^{14\pm1}$	163±3	$4\times10^{13\pm2}$	150±6	$6\times10^{12\pm4}$
0.7	168±6	$1\times10^{13\pm3}$	174±14	$1\times10^{14\pm21}$	169±3	$4\times10^{15\pm2}$	173±4	$2\times10^{14\pm2}$	184±5	$4\times10^{15\pm3}$
0.8	176±5	$5\times10^{13\pm3}$	201±12	$1\times10^{16\pm13}$	248±6	$2\times10^{20\pm3}$	277±9	$8\times10^{22\pm6}$	306±13	$2\times10^{25\pm8}$
0.9	310±11	$3\times10^{23\pm8}$	315±15	$5\times10^{21\pm4}$	321±19	$1\times10^{24\pm33}$	352±15	$4\times10^{26\pm16}$	396±19	$9\times10^{29\pm20}$
**KAS**										
0.1	122±11	$8\times10^{11\pm14}$	119±14	$2\times10^{11\pm32}$	115±15	$6\times10^{13\pm4}$	110±7	$4\times10^{13\pm5}$	82±14	$3\times10^{8\pm36}$
0.2	151±8	$4\times10^{13\pm5}$	150±10	$9\times10^{13\pm8}$	148±12	$4\times10^{14\pm17}$	138±9	$5\times10^{13\pm7}$	118±9	$2\times10^{11\pm8}$
0.3	159±6	$6\times10^{13\pm3}$	157±8	$1\times10^{14\pm6}$	153±8	$2\times10^{14\pm5}$	148±7	$6\times10^{13\pm5}$	126±9	$5\times10^{11\pm7}$
0.4	163±7	$3\times10^{13\pm4}$	162±8	$1\times10^{14\pm5}$	160±6	$1\times10^{14\pm3}$	155±5	$6\times10^{13\pm3}$	133±8	$1\times10^{12\pm6}$

0.5	164±9	$9\times10^{12\pm6}$	163±8	$8\times10^{13\pm5}$	162±3	$1\times10^{14\pm2}$	158±4	$6\times10^{13\pm2}$	139±8	$2\times10^{12\pm5}$
0.6	164±9	$9\times10^{12\pm6}$	165±6	$6\times10^{13\pm3}$	164±1	$7\times10^{14\pm1}$	161±4	$7\times10^{13\pm2}$	148±7	$1\times10^{13\pm4}$
0.7	166±6	$2\times10^{13\pm4}$	167±4	$7\times10^{13\pm2}$	169±3	$8\times10^{15\pm2}$	172±4	$4\times10^{14\pm2}$	183±6	$7\times10^{15\pm3}$
0.8	175±6	$7\times10^{13\pm3}$	197±10	$1\times10^{16\pm7}$	251±7	$6\times10^{20\pm3}$	281±9	$2\times10^{23\pm6}$	311±15	$5\times10^{25\pm8}$
0.9	315±11	$8\times10^{23\pm8}$	328±17	$3\times10^{25\pm15}$	336±10	$3\times10^{20\pm33}$	359±15	$1\times10^{27\pm16}$	405±10	$2\times10^{30\pm20}$

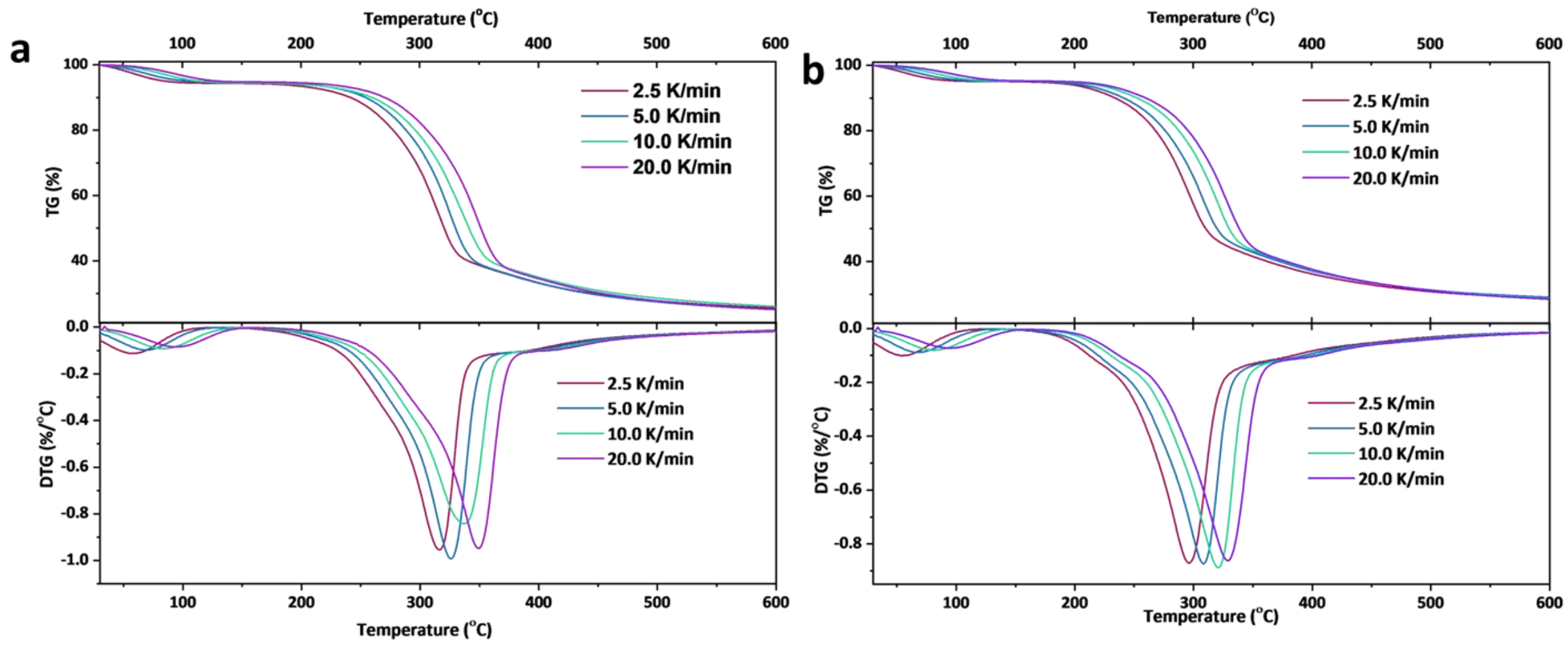

Figure S1. Thermal decomposition behavior of 0.2%NaOH+PW (a) and 1.0%NaOH+PW (b) at heating rates of 2.5, 5.0, 10.0, and 20.0 K/min, showing the stages of dehydration, devolatilization, and biochar formation.

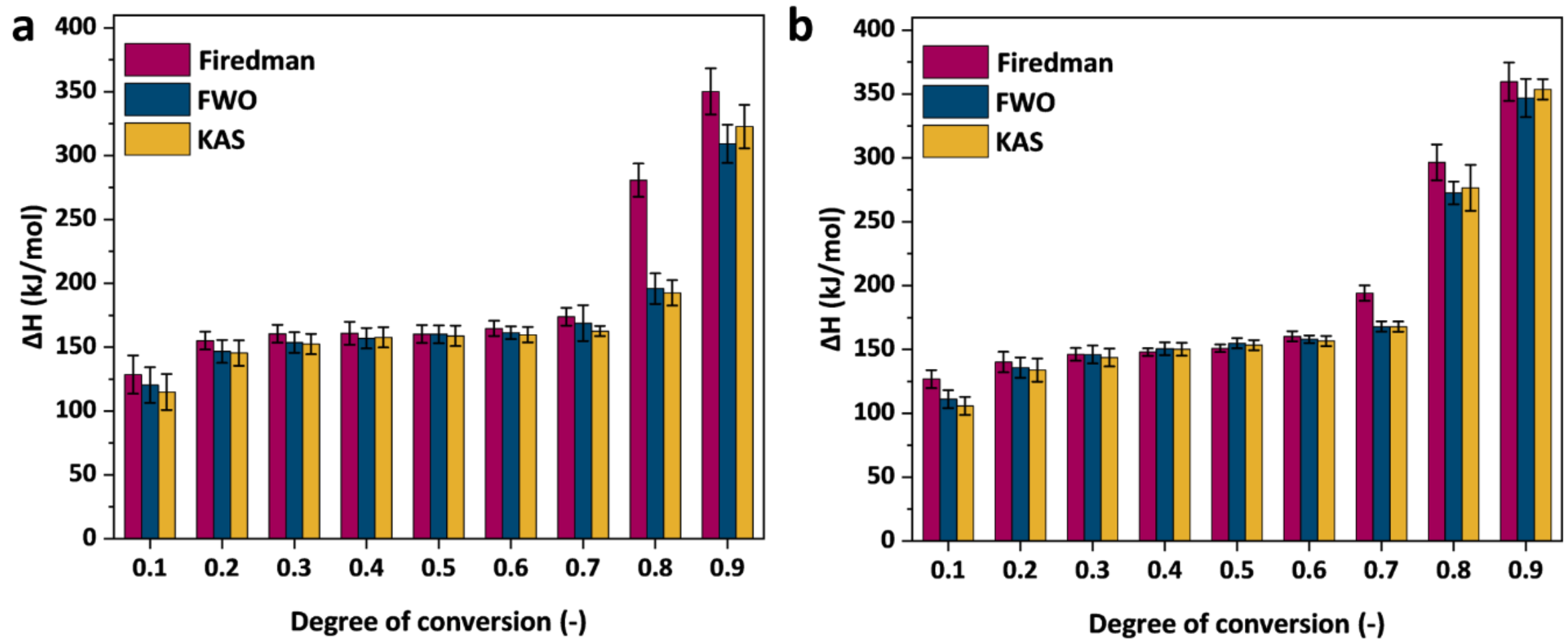

Figure S2. Enthalpy (ΔH) changes with respect to the degree of conversion (α) calculated using the Friedman, FWO, and KAS models for 0.2%NaOH+PW (a) and 1.0%NaOH+PW (b).

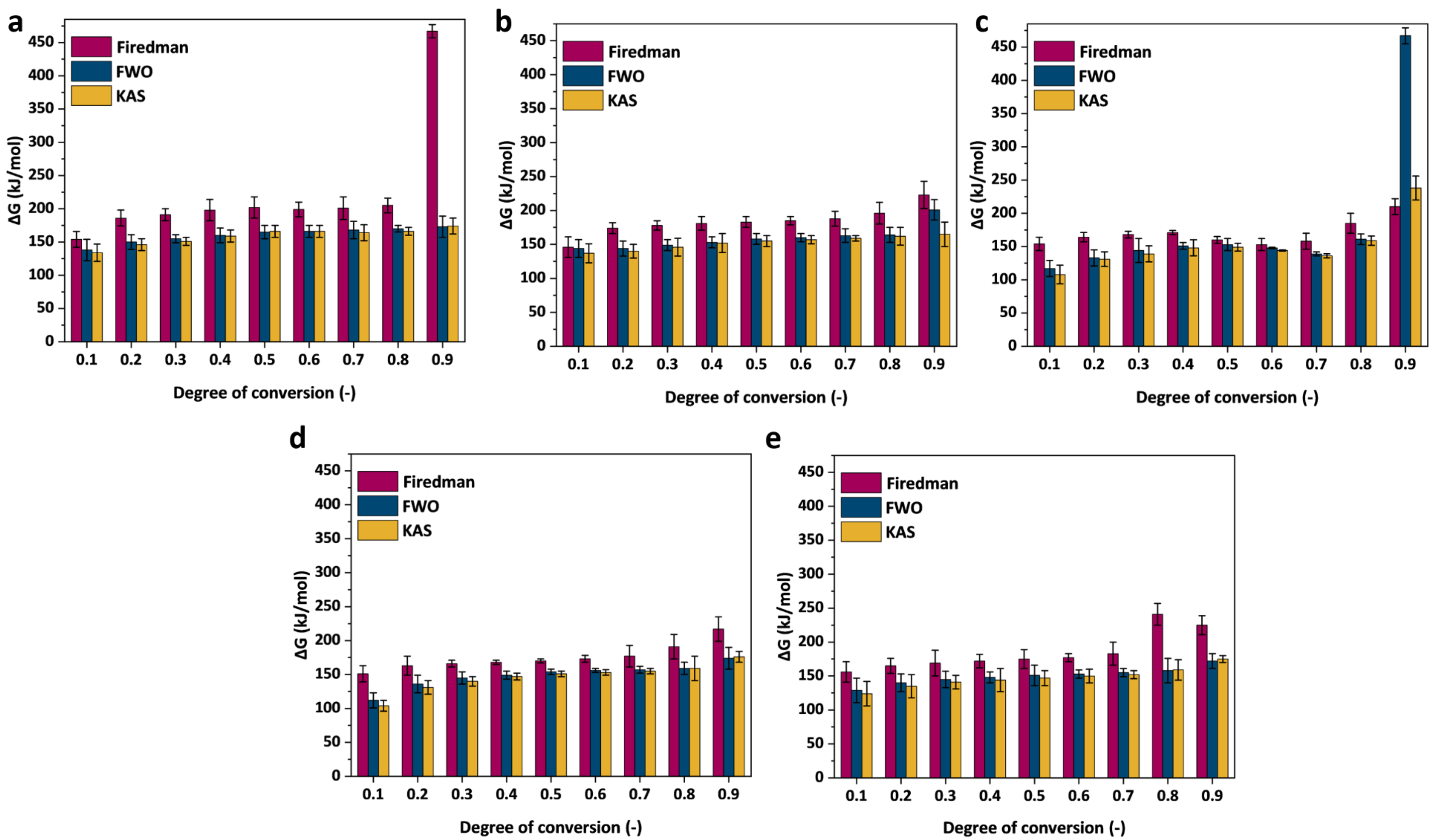

Figure S3. Gibbs free energy (ΔG) changes with respect to the degree of conversion (α) calculated using the Friedman, FWO, and KAS models for 0%NaOH+PW (a), 0.2%NaOH+PW (b), 0.5%NaOH+PW (c), 1.0%NaOH+PW (d) and 2.0%NaOH+PW (e).

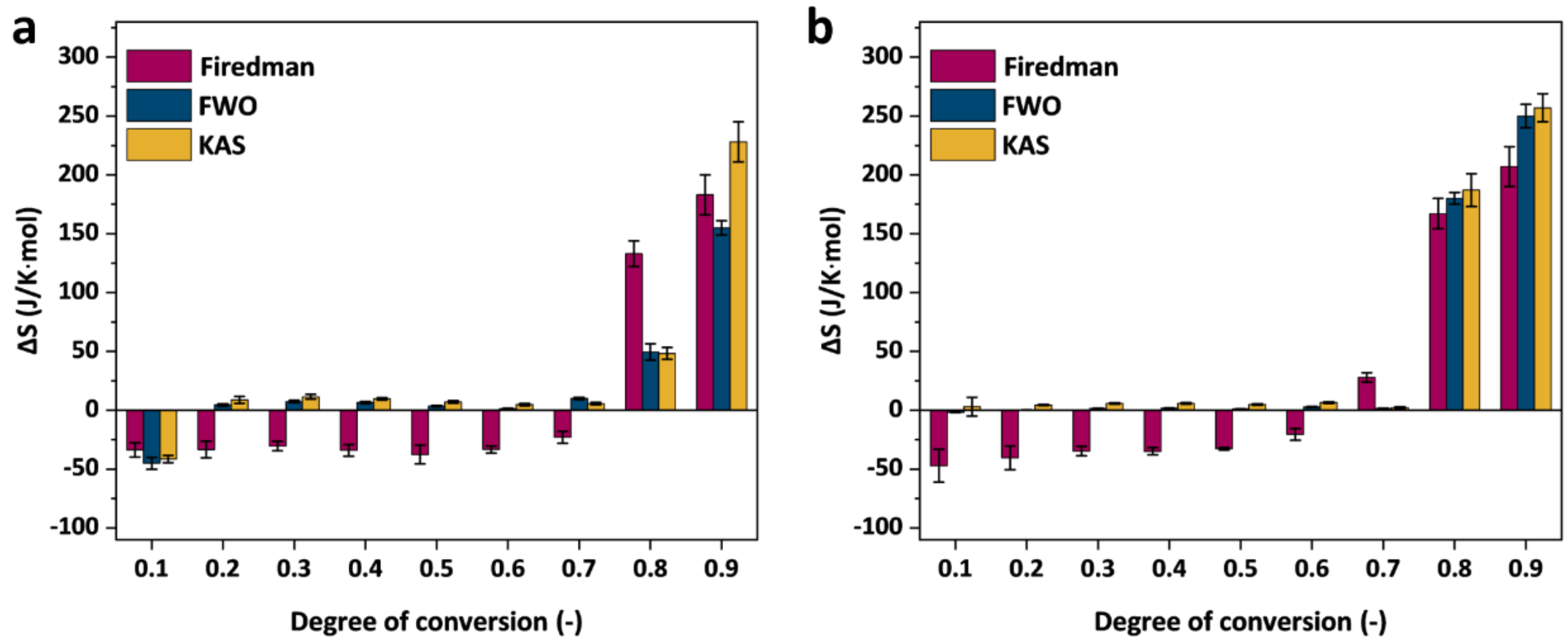

Figure S4. Entropy (ΔS) changes with respect to the degree of conversion (α) calculated using the Friedman, FWO, and KAS models for 0.2%NaOH+PW (a) and 1.0%NaOH+PW (b).
